# *Spa* Diversity among MRSA and MSSA Strains of *Staphylococcus aureus* in North of Iran

**DOI:** 10.1155/2010/351397

**Published:** 2010-08-31

**Authors:** Fatemeh Shakeri, Abolfath Shojai, Masoud Golalipour, Somaye Rahimi Alang, Hamid Vaez, Ezzat Allah Ghaemi

**Affiliations:** ^1^Islamic Azad University of Lahijan, Lahijan, Iran; ^2^Basic Sciences Faculty, Golestan University, Gorgan, Iran; ^3^Golestan University of Medical Sciences, Gorgon 4934174515, Iran; ^4^Microbiology Department, Infectious Disease Research Center, Gorgan, Iran

## Abstract

Protein A of *Staphylococcus aureus* is a pathogenic factor whose encoding gene, *spa*, shows a variation in length in different strains. In this study the *spa* gene variation in *S. aureus* isolated from healthy carriers and patients was studied, We also compared this variation among MRSA with MSSA strains. 208 strains of *Staphylococcus aureus* which we were isolated from Gorgan, north of Iran were studied, 121 cases from patients and 87 cases from healthy carriers, 59 out of them were *MRSA* and 149 *MSSA*. Samples DNA were extracted and amplified by specific primer of *spa* gene. In 4 (3.8%) strains of them no *spa* gene was detected, and 10.6% had a dual band (1200 and 1400 bp). In strains with one band, the length of *spa* gene differed from 1150 to 1500 bp. The most prevalent length was 1350–1400 bp (37%). The frequencies of short *spa* bands (1150–1200 bp) in patients strains were significantly higher. In 4 (3.8%) strains of them no *spa* gene was detected, and 10.6% had a dual band (1200 and 1400 bp). In strains with one band, the length of *spa* gene differed from 1150 to 1500 bp. The most prevalent length was 1350–1400 bp (37%). The frequencies of short *spa* bands (1150–1200 bp) in patients strains were significantly higher. The *spa* gene length of 1350–1400 bp in MSSA was more than in MRSA strains (*P* < .05). The average length of *spa* in isolated strains from urinary tract infections was more than others. It is concluded that the length of *spa* gene depends either on resistance to Methicillin or the source of *S. aureus* isolation.

## 1. Introduction

*Staphylococcus aureus *is one of the most important infectious pathogens in either hospitals or within the community. Protein A is a virulence factor with molecular weight of 42 KD [1]. It is covalently anchored to the peptidoglycan of *S. aureus*. 90% of protein A is found in the cell wall and the remaining 10% is free in the cytoplasm of bacteria. In some strains of *S. aureus*, protein A is unable to adhere to the cell wall and therefore is released into the media (secretary protein). This is mainly seen among meticillin-resistant *S. aureus* (MRSA) strains [2].

Protein A is an antiphagocytic protein that is based on its ability to bind the Fc portion of immunoglobulin G (IgG). Its NH2-terminal part contains five homologous IgG-binding units, A, B, C, D, and E, consisting of approximately 58 amino acids each. The COOH-terminal part of this protein which is thought to bind to the cell wall of *Staphylococcus aureus *consists of several repeats of an octapeptide. This protein acts as antiplatelet, anticomplement, and mytogen, [1, 3]. It is also presented as antigen and can be detected by specific antibody in rapid diagnostic test. 

Protein A has been coded by *spa* gene. In *spa* gene, the repeated part is located at 3′ end and identified as X region; the repetitive part of region X consists of up to 12 units each with a length of 24 nucleotides. This 24 nucleotides region is highly polymorphic with respect to the number and sequence of repeats. Diversity of X region causes variation in different protein A *Staphylococcus aureus* [4, 5]. Strain typing of *Staphylococcus aureus* is a good tool for epidemiologic purpose, and many genotypic and phenotypic techniques are used to apply that. Protein A Gene, due to X repeatable area, is considered as a good one. In this research, the diversity of *spa* gene in *staphylococcus aureus* isolated from patients and healthy carriers in this region was established and their diversity among MRSA and MSSA isolates were compared.

## 2. Material and Method

### 2.1. Sample Collection and Identification

The sample population in this study consists of 208 isolates of *Staphylococcus aureus* which were collected from 87 (41.8%) cases of health care workers from Gorgan central hospitals located in the north of Iran, and 121 cases (58.2%) of patients which referred to different medical laboratories in Gorgan during 2009.

 The primary diagnosis of *S. aureus *based on bacterial growth on Manitol Salt Agar media, Gram Stainning, Catalase, slide or tube Coagulase and Dnase test. We used the specific primers for glutamate synthetase gene (Table 1) to confirm the bacterial diagnosis [6].

*S. aureus *resistance to methicillin was determined on the base of presence of *mecA* gene, using specific primers (Table 1); the amplicon size was 533 bp [7, 8]. According to this method we found that from 208 isolated *S. aureus*, 59 (28.4%) and 149 (71.6*%*) were MRSA and MSSA, respectively (Unpublished data from our laboratory).

### 2.2. spa Typing

 Genomic DNA for subsequent PCR was isolated from 1-ml overnight culture lysed with lysozym-phenol chloroform method and treated with N-lauroyl sarcosine sodium salt 2% (300 *μ*L), proteinase k 100 *μ*g (30 *μ*l), and RNase A(5 *μ*l). DNA was extracted by phenol chloroform isoamilalcohol, chloroform, and cold ethanol [9, 10].

For *spa* gene PCR, primer showed in Table 1 was used to identify the whole protein A genome [11].

The PCR mixture consisted of 1 mmol/L magnesiumchloride, 0.2 mmol/L dNTPs, PCR buffer, 1 *μ*mol/L of primers, and 1 unit of Taq-DNA polymerase in a final volume of 50 *μ*L. Samples were denaturated at 94°C for 4 minutes followed by 35 cycles using the following parameters: denaturation at 94°C for 1 minute, annealing at 56°C for 1 minute, and extension at 72°C for 3 minutes, with a final extension at 72°C for 5 minutes [4]. In this study COL strain was used as a positive (precinct 1200 bp in *spa* band), and distill water as negative control. Data were entered in spss software version 16 and analyzed with X^2^ and Anova tests, and *P* value of <  .05 was considered as significant.

## 3. Result

**Figure 1:**
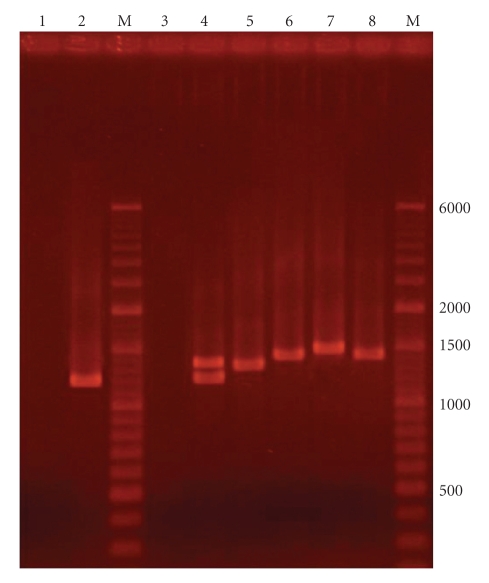
The pattern of bands of *spa* gene in *S. aureus *isolated in north of Iran. M: mark for DNA Ladder (6000 bp with a 100 bp to 1500 bp, Company Fermentas). Number 1 is negative and 2 positive control (Col strain), respectively. Number 3 and 4 are the strains *of Staphylococcus aureus* without *spa* band and two bands of *spa* gene and numbers 5–8 are strains *of Staphylococcus aureus* with one band of *spa* gene.

 We did not find the *spa* gene band in eight (3.8%) tested *S. aureus*. In 22 (10.6%) cases double bands of *spa* gene were observed with the length of 1200 and 1400 bp (Figure 1) and the reminding 179 cases (86.1%) had one band.

The frequency of *S. aureus *isolates with no band, one band, and two bands of *spa* gene, in healthy carriers, was 5.8%, 88.4%, and 5.8%, and in patients, 2.5%, 83.5%, and 14%, respectively (*P* = .088). But the frequency of strains having two bands in patients are significantly more than carriers (*P* = .042). Three strains of *S. aureus *without *spa* gene were isolated from patients, and the urine was the source of these bacteria and this phenomenon were not observed in any other clinical sample.

 The most common *spa* gene length in *S. aureus *strains was about 1350–1400 bp (37%), but frequency of strains which had short band (1150–1200 bp) in patients were significantly more than healthy carriers (20.7% versus 4.6%) (*P* = .003) (Table 2).

The frequency of *S. aureus *isolates with no band, one band, and two bands of *spa* gene, in MRSA isolates was 2 (3.4%), 49 (83%), and 8 (13.6%) cases, and in MSSA strains was 6 (4%), 129 (86.6%), and 14 (9.4%) cases, respectively.

Majority of MRSA strains that have one band of protein A gene, have the length band between 1150–1200 bp, while most frequencies in MSSA strains have the length band between 1350–1400 bp. The differences in length is significant between two groups of MRSA and MSSA (*P* < .001) (Table 3).

The Prevalence of strains with two bands of *spa* gene in isolated from wounds (21.4%) and urine (16.7%) was more than in other clinical samples, and the most cases with a short band of *spa* gene (1150–1200 bp) were separated from wounds and blood infection. According to Table 4, the average length of *spa* gene in strains which were isolated from urinary tract infections is more than other strains.

The average ages of people who were infected with *S. aureus *isolates which consisted of two bands, one band, and no band were 17.4 ± 30.2, 19.5 ± 32.8, and 8.4 ± 35.5 years, respectively, but this difference is not meaningful statistically (*P* > .05). There is no statistical difference between the *spa* gene types among *S. aureus *isolated from males and females (*P* > .05).

## 4. Discussion

In this study, 8 isolates of *Staphylococcus aureus *(3.8%) had no *spa* gene band, and most of them were observed in strains which were isolated from healthy carriers; therefore frequency of protein A gene was about 96.2% (96.6% in MRSA versus 96% in MSSA). In other studies, the prevalence of *S. aureus *without protein A expression is reported to be up to 5% [12], but different studies on *spa* gene showed a lower frequency of strains without this gene. Faria and colleagues showed that 99% of *Staphylococcus aureus *strains can be typed with *spa* gene [13]. Also Strommenger showed that in 1459 strains of Staphylococcus aureus, 99.8% strains are typeable by *spa* gene typing [11] these later findings seem to be lower than our results. We could not find any documented report in the literature working with PCR method on *spa* gene and demonstrating gene with double bands. Our study is the first documented report of the strains of *S. aureus *with two *spa* gene. The frequency of this phenomenon is 10.6% with the length of 1200 and 1400 bp. 

In addition to standard diagnostic methods we tested all *S. aureus *isolates by specific glutamate synthetase (*sa442*); we also carried out the *spa* gene PCR method in triple times and identical results were found; due to this observation we argue strongly in the favor of new strain of *S. aureus *in our regions. This strain is more prevalent in patients than healthy carriers and it is more common in MRSA than MSSA. The more accurate identification of this strain and its importance is necessary.

Our study has showed that the length of *spa* gene in MRSA strains was significantly shorter than MSSA strains. This issue indicated that the number of repetitive sequences in Xr *spa* in MSSA strains is more than in MRSA strains. Frequencies of strains with short *spa* bands (1150–1200 bp) in strains isolated from patients were significantly more than from healthy carriers. This can be due to the requirement of bacterial attachment to the nasal epithelial cells in healthy carriers by a longer protein A. We argue that *S. aureus *strains with shorter length of protein A cannot adhere to the surface of nasal epithelium and is discharged by breath, sneezing, and coughing [14].

Protein A has a recognized pathogenic role in *S. aureus*, and this process is via the anchoring to Fc Domain IgG, Complement fixation, and so forth. This protein also has a role in attachment and invasion of target cells, joung and colleagues (2001) show the role of this protein in KB cells connection [15].

Although the length of *spa* gene do not have a meaning correlation with the type of infection; our result showed that the length of *spa* gene in strains which are isolated from urinary tract infection is more than samples from blood, wounds, and other clinical samples. This observation may be due to the role this protein plays in conjunction or stronger connection of bacteria to the urinary tract epithelial cells [16].

## 5. Conclusion

Our study showed that 3.8% of *Staphylococcus aureus *strains in Gorgan (north of Iran) have no *spa* genes. About 10% of them were consisted of dual-band *spa* in PCR. The majority of our strains showed to have the length of *spa* gene between 1350–1400 bp. Frequencies of strains with short *spa* bands (1150–1200 bp) in strains isolated from patients were significantly more than those isolated from healthy carriers. The average length of *spa* gene in strains which were isolated from urinary tract infections was more than other clinical strains. It is concluded that the length of *spa* gene depends either on resistance to methicillin or the source of *S. aureus * isolation.

## Figures and Tables

**Table 1:** The genes and related primers used in this study.

Gene		Primers	Amplicon size
*sa442 *	F	5-CGTAATGAGATTTCAGTAGATAATACAACA-3	108 bp
	R	5-AATCTTTGTCGGTACACGATATTCTTCACG-3	
*mec A*	F	5-AAAATCGATGGTAAAGGTTGGC-3	533 bp
	R	5-AGTTCTGCAGTACCGGATTTGC-3	
*spa*	F	5- ATCTGGTGGCGTAACACCTG-3	Variable:1150–1500 bp
	R	5- C GCTGCACCTAACGCTAATG-3	

**Table 2:** Distribution of *spa* gene in *S. aureus* strains according their source.

	Source of isolation bacteria		
	Healthy carriers	Patients	Total
Size and type of *spa* band			
No band	5 (5.8%)	3 (2.5%)	8 (3.8%)
Two band	5 (5.8%)	17 (14%)	22 (10.6%)

One band			
1150–1200	4 (4.6%)	25 (20.7%)	29 (13.9%)
1250–1300	7 (8.1%)	10 (8.3%)	17 (8.2%)
1350–1400	35 (40.1%)	42 (34.7%)	77 (37%)
1450–1500	31 (35.6%)	24 (19.8%)	55 (26.4%)

Total	87 (100%)	121 (100%)	208 (100%)

**Table 3:** Distribution of *spa* gene in MRSA and MSSA isolates in Gorgan, north of Iran.

	Type of *S. aureus *		
	MRSA	MSSA	Total
Size and type of *spa* band			
No band	2 (3.4%)	6 (4%)	8 (3.8%)
Two band	8 (13.6%)	14 (9.4%)	22 (10.5%)

One band			
1150–1200	20 (33.9%)	9 (6.1%)	29 (13.9%)
1250–1300	3 (5.1%)	14 (9.4%)	17 (8.1%)
1350–1400	15 (25.4%)	62 (41.6%)	77 (37%)
1450–1500	11 (18.6%)	44 (29.5%)	55 (26.4%)

Total	59 (100%)	149 (100%)	208 (100%)

**Table 4:** Average length of *spa* gene in *Staphylococcus aureus* isolated from patient in Gorgan, north of Iran.

Sample	Number of isolated *S. aureus *	Average size of *spa *(bp)	SD (±)
Urine	32	1375.56	81.304
Wound	22	1304.55	120.425
Blood	24	1318.75	103.012
Other	13	1342.31	101.748

Total	91	1339.01	103.226

## References

[1] Palmqvist N, Foster T, Tarkowski A, Josefsson E (2002). Protein A is a virulence factor in *Staphylococcus aureus* arthritis and septic death. *Journal of Microbial Pathogenesis*.

[2] Movitz J (1974). A study on the biosynthesis of protein A in *Staphylococcus aureus*. *European Journal of Biochemistry*.

[3] Uhlen M, Guss B, Nilsson B (1984). Complete sequence of the staphylococcal gene encoding protein A. A gene evolved through multiple duplications. *Journal of Biological Chemistry*.

[4] Wichelhaus TA, Hunfeld K-P, Böddinghaus B, Kraiczy P, Schäfer V, Brade V (2001). Rapid molecular typing of methicillin-resistant *Staphylococcus aureus* by PCR-RFLP. *Infection Control and Hospital Epidemiology*.

[5] Mitani N, Koizumi A, Sano R (2005). Molecular typing on methicillin-resistant *Staphylococcus aureus* by PCR-RFLP and its usefulness in an epidemiological study of an outbreak. *Japanese Journal of Infectious Diseases*.

[6] Samadi N, Alvandi M, Fazeli MR, Azizi E, Mehrgan H, Naseri M (2007). PCR-based detection of low levels of *Staphylococcus aureus* contamination in pharmaceutical preparations. *Journal of Biological Sciences*.

[7] Louie L, Goodfellow J, Mathieu P, Glatt A, Louie M, Simor AE (2002). Rapid detection of methicillin-resistant staphylococci from blood culture bottles by using a multiplex PCR assay. *Journal of Clinical Microbiology*.

[8] Louie L, Matsumura SO, Choi E, Louie M, Simor AE (2000). Evaluation of three rapid methods for detection of methicillin resistance in *Staphylococcus aureus*. *Journal of Clinical Microbiology*.

[9] Mehndiratta P, Bhalla P, Ahmed A, Sharma Y (2009). Molecular typing of methicillin-resistant *Staphylococcus aureus* strains by PCR-RFLP of *spa* gene. *Indian Journal of Medical Microbiology*.

[10] Nimmo GR, Bell GM, Mitchel D, Gosbell LA, Perman JW, Turnidge JD (2003). Anti microbal resistance in *Staphylococcus aureus* in australian teaching hospital. *Journal of Microbial Drug Resistance*.

[11] Strommenger B, Braulke C, Heuck D (2008). *spa* typing of *Staphylococcus aureus* as a frontline tool in epidemiological typing. *Journal of Clinical Microbiology*.

[12] Adesida SA, Likhoshvay Y, Eisner W (2006). Repeats in the 3’ region of the protein A gene is uniquein a strain of *Staphylococcus aureus* recovered from wound infections in Lagos,Nigeria. *African Journal of Biotechnology*.

[13] Faria NA, Carrico JA, Oliveira DC, Ramirez M, De Lencastre H (2008). Analysis of typing methods for epidemiological surveillance of both methicillin-resistant and methicillin-susceptible *Staphylococcus aureus* strains. *Journal of Clinical Microbiology*.

[14] Fenner L, Widmer AF, Dangel M, Frei R (2008). Distribution of *spa* types among meticillin-resistant *Staphylococcus aureus* isolates during a 6 year period at a low-prevalence university hospital. *Journal of Medical Microbiology*.

[15] Jung KY, Cha JD, Lee SH (2001). Involvment of staphylococcal protein A and cytoskeletal action in *Staphylococcus aureus* invasion of cultured human oral epithelial cell. *Journal of Medical Microbiology*.

[16] Jung KY, Cha JD, Lee SH (2001). Involvment of staphylococcal protein A and cytoskeletal action in *Staphylococcus aureus* invasion of cultured human oral epithelial cell. *Journal of Medical Microbiology*.

